# LONGITUDINAL ASSOCIATIONS BETWEEN MULTIMORBIDITIES AND PATIENT-REPORTED QUALITY OF LIFE

**DOI:** 10.1093/geroni/igae098.2176

**Published:** 2024-12-31

**Authors:** Eileen Graham, Olivia Atherton, Daniel Mroczek, Marquita Lewis Thames, Michael Wolf

**Affiliations:** Northwestern University, Evanston, Illinois, United States; University of Houston, Houston, Texas, United States; Northwestern University, Evanston, Illinois, United States; Northwestern University, Evanston, Illinois, United States; Northwestern University, Evanston, Illinois, United States

## Abstract

The global prevalence of multimorbidity is increasing as the population ages. Older adults are very likely to develop multiple chronic conditions, and nearly two-thirds of the older adult population in the U.S. are estimated to be experiencing two or more chronic conditions. The current study examined whether multimorbidity was associated with longitudinal patterns in health-related quality of life (i.e., anxiety, depression, physical function) and whether these associations were moderated by sociodemographic factors (i.e., sex, race, marital status, income, insurance, education). The present pre-registered study used data from the Health Literacy and Cognitive Function Among Older Adults Longitudinal Study (LitCog), a prospective cohort study of English-speaking older adults (N=900). At each measurement occasion, participants reported anxiety, depression, and physical function using the Patient Reported Outcomes Information System (PROMIS), current chronic conditions, and socio-demographic characteristics. We employed multilevel growth models to estimate changes in health-related quality of life, with multimorbidities as a predictor and socio-demographics as covariates. Results indicated that individuals with multiple chronic conditions reported persistently high levels of anxiety and depression, and worse physical function. We found evidence for racial health disparities, such that individuals who identified as non-white experienced worsening health-related quality of life as multimorbidities increased, relative to white participants. These results contribute to the current conversation about the long term impacts of structural and systemic barriers experienced by minoritized groups. We further discuss the public health implications of multimorbidity in older adulthood.

